# Poster Session I - A53 COMPUTER-AIDED COLONOSCOPY ALERT FATIGUE AND ITS EFFECT ON ADENOMA DETECTION

**DOI:** 10.1093/jcag/gwaf042.053

**Published:** 2026-02-13

**Authors:** F Huang, V Michal, R Djinbachian, M Oleksiw, D K Rex, R Battat, E deslandres, M Bouin, J Liu, B Panzini, S Bouchard, D C Daoud, E Bernard, K Orlicka, R Leduc, L D’aoust, D von Renteln

**Affiliations:** Universite de Montreal Faculte de Medecine, Montreal, QC, Canada; Centre de Recherche du Centre Hospitalier de l’Universite de Montreal, Montreal, QC, Canada; Centre Hospitalier de l’Universite de Montreal, Montreal, QC, Canada; Universite de Montreal Faculte de Medecine, Montreal, QC, Canada; Indiana University School of Medicine, Indianapolis, IN; Centre Hospitalier de l’Universite de Montreal, Montreal, QC, Canada; Centre Hospitalier de l’Universite de Montreal, Montreal, QC, Canada; Centre Hospitalier de l’Universite de Montreal, Montreal, QC, Canada; Centre Hospitalier de l’Universite de Montreal, Montreal, QC, Canada; Centre Hospitalier de l’Universite de Montreal, Montreal, QC, Canada; Centre Hospitalier de l’Universite de Montreal, Montreal, QC, Canada; Centre Hospitalier de l’Universite de Montreal, Montreal, QC, Canada; Centre Hospitalier de l’Universite de Montreal, Montreal, QC, Canada; Centre Hospitalier de l’Universite de Montreal, Montreal, QC, Canada; Centre Hospitalier de l’Universite de Montreal, Montreal, QC, Canada; Centre Hospitalier de l’Universite de Montreal, Montreal, QC, Canada; Centre Hospitalier de l’Universite de Montreal, Montreal, QC, Canada

## Abstract

**Background:**

Computer-aided detection (CADe) can improve colonoscopy adenoma detection rates (ADR). However, prolonged exposure to repeated alerts may lead to alert fatigue, potentially diminishing its effectiveness.

**Aims:**

We aimed to determine whether alert fatigue occurs during CADe use and, if so, how it influences ADR throughout the day.

**Methods:**

We conducted a secondary analysis of a prospectively collected cohort to evaluate how ADR varied with and without CADe in a pragmatic, real-world implementation. Elective colonoscopies performed at the University of Montreal Hospital Center were analyzed according to their position within the endoscopist’s daily schedule. The primary outcome was ADR across procedure order, adjusted for withdrawal time (WT). Secondary outcomes included adenomas per colonoscopy (APC), ADR by time of day, and ADR across different WT categories.

**Results:**

A total of 1,700 colonoscopies were analyzed, including 880 with CADe assistance and 820 without. In the CADe-unassisted group, ADR remained stable throughout the day (1st–3rd procedures: 39.4%, 95% CI [35.1–43.8]; 4th–7th procedures: 40.9%, 95% CI [32.9-49.5]). By contrast, ADR in CADe-assisted procedures was markedly higher in the first three procedures (49.9%, 95% CI [45.5–54.3]) but dropped significantly to 39.9% (95% CI [33.8–46.3]) in later procedures. This pattern was consistent when ADR was assessed by time of day. Similarly, APC were significantly higher in early CADe-assisted procedures (mean increase +0.27, 95% CI [0.14–0.41], p < 0.001), but this benefit disappeared in later procedures (+0.11, 95% CI [–0.09–0.31], p = 0.51). WT analysis further supported this effect: ADR gains per additional minute were higher in unassisted colonoscopies (21.6% early vs. 15.3% late) than in CADe-assisted colonoscopies (10.5% early vs. 10.1% late).

**Conclusions:**

CADe assistance significantly improved ADR during early-day colonoscopies, but this benefit disappeared in later-day procedures. This pattern suggests that alert fatigue may limit endoscopists’ responsiveness to CADe prompts as the day progresses.

**Funding Agencies:**

None

